# Rotating Night Shifts and Physical Well-Being in Nurses: Cross-Sectional Associations Consistent with a Sleep Quality Pathway

**DOI:** 10.3390/nursrep16010019

**Published:** 2026-01-08

**Authors:** Andreja Kolarić, Azeem Majeed, Mate Car, Ivan Miskulin

**Affiliations:** 1 Faculty of Health Sciences, Catholic University of Croatia, 10000 Zagreb, Croatia; 2 Clinical Hospital Merkur, 10000 Zagreb, Croatia; 3 School of Public Health, Imperial College London, London SW7 2AZ, UK; 4 Centre for Data-Driven Policy and Management, Catholic University of Croatia, 10000 Zagreb, Croatia; 5 Faculty of Medicine Osijek, Josip Juraj Strossmayer University of Osijek, 31000 Osijek, Croatia

**Keywords:** shift work, circadian disruption, sleep quality, rotating night shifts, nurses, physical health, mediation analysis, circadian burden

## Abstract

**Background**: Rotating and night-including shifts disrupt circadian alignment, impair sleep, and may reduce nurses’ physiological recovery. **Objectives**: This study aimed (1) to compare sleep quality and physical well-being across four shift schedules among hospital nurses and (2) to examine whether the association between rotating shifts and physical well-being was statistically consistent with an indirect association via sleep quality. **Methods**: In this cross-sectional study, 173 nurses from a tertiary hospital in Zagreb, Croatia, completed validated measures of sleep quality and physical well-being. Four shift patterns were analyzed—fixed morning, morning–afternoon, extended 12-h, and rotating three-shift—using Welch ANOVA and regression models. A bootstrapped mediation analysis (10,000 resamples; BCa method), interpreted as a statistical decomposition, estimated an indirect association consistent with sleep quality. **Results**: Rotating-shift nurses reported the poorest sleep (PSQI = 10.2 ± 2.6; *p* = 0.003). Physical well-being did not differ significantly across shift types (*p* = 0.08), although rotating-shift nurses had the lowest mean physical scores (24.3 ± 4.4). The rotating-shift subgroup was small (n = 16), limiting precision. The mediation analysis was statistically consistent with an indirect association between rotating shifts and physical well-being via sleep quality (ACME = −1.85, 95% CI −3.05 to −0.88; *p* < 0.001), while the proportion of the total association was imprecisely estimated. **Conclusions**: In this single-site cross-sectional sample, rotating night shifts were associated with poorer sleep and, on average, lower physical well-being; patterns were statistically consistent with an indirect association via sleep quality. Because exposure, mediator, and outcome were measured concurrently, these findings are hypothesis-generating and do not establish causality.

## 1. Introduction

Disruption of the circadian system is one of the most pervasive biological consequences of modern healthcare work. Continuous hospital services require staffing beyond daylight hours, exposing nurses—the largest group of healthcare professionals—to rotating and night-including schedules that desynchronize the endogenous circadian rhythm from the external light–dark cycle [1,2]. Such misalignment alters melatonin secretion, core body temperature rhythms, and sleep architecture, contributing to metabolic and cardiovascular strain as well as impaired recovery [3,4,5]. Rotating and night schedules are consistently associated with poorer sleep quality among nurses, often measured with Pittsburgh Sleep Quality Index (PSQI), and with elevated symptoms of shift-work disorder and related health risks [6,7,8]. Beyond sleep quality per se, shift schedules have been linked to diminished on-duty alertness and to adverse cardiometabolic profiles in hospital staff [8,9,10].

Shift work therefore represents both a social and a biological stressor. While conventional daytime schedules support stable sleep–wake cycles, non-standard arrangements—night duties, extended 12-h shifts, weekend work, and on-call patterns—disrupt the synchronization between circadian timing and behavioral demands [11]. Approximately one-third of healthcare workers worldwide are engaged in shift work [1], with nurses most commonly alternating between morning, afternoon, and night duties. The consequences include poor sleep quality, reduced alertness, fatigue, and diminished quality of life [12,13].

Despite extensive literature on the adverse outcomes of shift work, few studies have quantified how much of the association between shift schedules and nurses’ well-being is statistically compatible with a pathway operating through degraded sleep quality rather than through direct occupational strain.

This study had two objectives: (1) to compare sleep quality and physical well-being (WHOQOL-BREF Physical Health domain) across four shift schedules among hospital nurses and (2) to examine whether associations between shift type and physical well-being were statistically consistent with an indirect association via sleep quality.

## 2. Materials and Methods

### 2.1. Study Design and Participants

This cross-sectional study was conducted at Clinical Hospital Merkur in Zagreb, Croatia, during 2023, and is reported in accordance with STROBE reporting guidelines.

**Figure 1:**
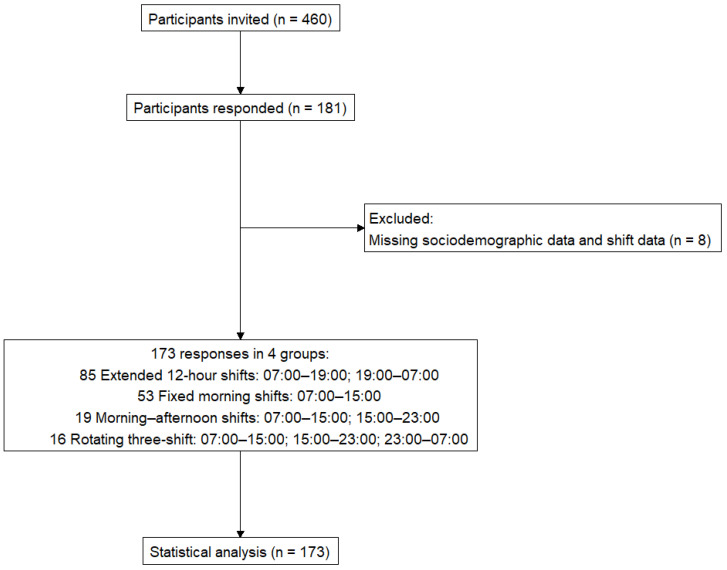
Presents the study design flowchart, outlining participant inclusion, exclusion, and classification into shift groups.

All 460 nurses employed at the hospital were invited to participate (single-site, voluntary response sample). Of these, 181 questionnaires were returned (response rate ≈ 39.3%), and 173 with complete sociodemographic data were included in the analysis (Figure 1).

Missing values were minimal (<5% per item). Analyses were performed on complete cases (N = 173).

### 2.2. Shift Classification

Participants self-reported their predominant shift pattern over the previous three months and were grouped as follows:

1. Fixed morning: 07:00–15:00.
2. Morning–afternoon: 07:00–15:00; 15:00–23:00 (no night hours).
3. Rotating three-shift: 07:00–15:00; 15:00–23:00; 23:00–07:00.
4. Extended 12-h: 07:00–19:00; 19:00–07:00.

On-call duty: On-call status was coded as a binary variable (yes/no) to capture additional workload/circadian burden not fully reflected by nominal shift categories and was included as a covariate in regression and mediation models.

### 2.3. Measures

Sociodemographic variables: Age, sex, education level, total years of nursing experience, and on-call status were recorded.

Sleep quality: The Croatian version of the Pittsburgh Sleep Quality Index (PSQI) (Buysse et al., 1989) [14] was used; scores 0–21 (higher = worse sleep). Internal consistency of the PSQI component scores was acceptable (Cronbach’s α = 0.68, 95% CI [0.60–0.75]; n = 173). This reliability is consistent with other studies using the PSQI in occupational and heterogeneous clinical samples, where α ≈ 0.65–0.75 reflects the multidimensional nature of the instrument rather than poor coherence.

Physical quality of life: The WHOQOL-BREF Physical Health domain (7–35, higher = better) measured physical well-being (mobility, energy, pain, daily activities) [15]. The Croatian version shows good reliability (α = 0.86); internal consistency was good (Cronbach’s α = 0.81, 95% CI [0.75–0.85]; n = 173).

### 2.4. Procedure

Head nurses distributed paper questionnaires (PSQI, WHOQOL-BREF, and sociodemographic items) to each ward. Participants had seven days to complete and return sealed forms to locked boxes. Two reminders were issued. No personal identifiers were collected. Data were collected between 19 April and 31 May 2023.

### 2.5. Statistical Analysis

All analyses were conducted in R (v4.5.2) using gtsummary (v2.4.0), ggstatsplot (v0.13.3), car (v3.1-3), and mediation (v4.5.1). Statistical significance was set at two-tailed α = 0.05. Residual normality and homoscedasticity were supported by Q–Q and residual–fitted plots (Figure S1). Data and full R scripts are publicly available at OSF (DOI: 10.17605/OSF.IO/8HCXU).

### 2.6. Descriptive Statistics

Participant characteristics were summarized as mean ± SD for continuous variables and n (%) for categorical variables, stratified by shift type. Between-group differences were examined using Welch’s ANOVA for continuous variables and *χ*^2^ or Fisher’s exact tests for categorical variables. Analyses used complete cases (N = 173); list-wise deletion was applied; no imputation.

### 2.7. Assumptions and Diagnostics

Model diagnostics included Q–Q and residual–fitted plots, Levene’s test for heteroscedasticity, variance inflation factors (VIF; for factors GVIF^(1/2Df)^) for multicollinearity, Durbin–Watson for residual autocorrelation, and influence statistics (Cook’s D, DFBETAS). Minor deviations from normality were acceptable given N = 173; we therefore complemented inference with nonparametric bootstrap confidence intervals (BCa, 10,000 resamples). Multicollinearity was low (all VIF < 5; GVIF^(1/2Df)^ < 2.5), Durbin–Watson statistics were ~2, and no influential observations exceeded Cook’s D ≥ 1 or |DFBETAS| ≥ 0.5; consequently, no case deletions were performed. Full diagnostics are shown in Figure S1.

### 2.8. Primary Analyses

To evaluate the association between shift work and sleep quality (PSQI), Welch’s ANOVA was used with post hoc Games–Howell comparisons.

A multiple linear regression was then fitted to predict WHOQOL-BREF Physical Health domain scores from shift type, sleep quality (PSQI), and covariates (age, education, work experience, on-call duty).

Standardized coefficients (β) and 95% confidence intervals were obtained via nonparametric bootstrap (10,000 resamples; BCa CIs).

To aid interpretation, we report standardized effect sizes—Hedges g for pairwise contrasts and ω^2^ for the omnibus—with 95% bootstrap confidence intervals for ω^2^ and standardized β for regression models. Although the rotating-shift group was relatively small (n = 16), the use of 10,000-resample bootstrapping helped quantify uncertainty under unequal group sizes; however, it does not eliminate the imprecision inherent to a small subgroup.

### 2.9. Mediation Analysis

Because the data are cross-sectional, this analysis does not establish mediation or causal pathways. We therefore use mediation terminology only to describe statistical decomposition, not temporal or causal processes.

A statistical mediation analysis (counterfactual-based, nonparametric bootstrap, 10,000 resamples) tested whether sleep quality was statistically consistent with an indirect association between rotating shifts and physical well-being.

We coded the exposure as rotating vs. other schedules (1 = rotating, 0 = other). We estimated two linear models with identical covariates (age, education, work experience, on-call). The mediator model was PSQI ~ rotating + covariates; the outcome model was WHOQOL-Physical ~ rotating + PSQI + covariates. Indirect (ACME), direct (ADE), and total effects were estimated using nonparametric bootstrap (10,000 resamples; BCa confidence intervals).

Results were summarized as: ACME (average mediation effect; labeled “Average Causal Mediation Effect” in the mediation package output), ADE (average direct effect), Total Effect, and Proportion Mediated, with 95% CIs and *p*-values. All variables were analyzed on their original scales to preserve interpretability; coefficients therefore represent unstandardized (raw-unit) effects.

Sensitivity to unmeasured confounding. Robustness of the indirect association was evaluated using the medsens function (mediation package), which varies the residual correlation (ρ) between mediator and outcome models from −1 to +1. The analysis identified the residual correlation and joint variance that an unmeasured confounder would need to explain to reduce the estimated ACME (indirect association) to zero. Visualization of ACME stability across ρ values and the contour of ACME = 0 across (R^2^_M, R^2^_Y) is provided in the Supplementary Materials (Figure S2).

### 2.10. Sensitivity and Robustness Checks

Two a priori robustness checks were performed to confirm that findings were not driven by group heterogeneity or scale effects.

First, all models were re-estimated after excluding participants with on-call duties (n = 19) to test whether their inclusion influenced the main associations. Results remained directionally similar, with the rotating-shift coefficient for PSQI persisting in the same direction (β = +2.05, *p* = 0.10).

Second, regression models were re-estimated with z-scored variables (mean = 0, SD = 1) to verify stability of coefficients and eliminate scale effects. Both checks produced comparable results, supporting the robustness of the primary findings.

### 2.11. Power and Sample Considerations

Rather than post hoc power calculations, we report effect sizes and 95% confidence intervals to convey the precision of all estimates.

The relatively small rotating-shift group limits the precision of the estimates; confidence intervals are therefore the most informative measure of evidential strength.

### 2.12. Ethical Considerations

Ethical approval was obtained from the Clinical Hospital Merkur Ethical Committee (ref. 0311-2994; 19 April 2023). All procedures were conducted in accordance with the Declaration of Helsinki and local regulations. Participation was voluntary and anonymous; nurses received written study information, and completion of the questionnaire was taken as implied informed consent.

## 3. Results

### 3.1. Participant Characteristics

Of the 460 nurses invited to participate, 181 returned questionnaires (response rate ≈ 39.3%), and 173 participants with complete sociodemographic data were included in the analyses (see Figure 1).

Participant demographics and main outcome scores are summarized in Table 1. On-call duty was reported by 19 nurses and was distributed unevenly across shift groups: morning shifts (n = 15) and rotating shifts (n = 4) included on-call responsibilities, whereas morning–afternoon and extended 12-h schedules did not include on-call duty (n = 0 in both groups). This pattern reflects routine staffing practices at the study site and motivated inclusion of on-call status as a covariate. Rotating-shift nurses tended to be younger and less experienced, which partly explains group variability and supports inclusion of these covariates in regression models.

Demographic patterns (younger and less-experienced nurses in rotating shifts) are typical of scheduling hierarchies in hospital systems and align with known predictors of circadian vulnerability.

FDR correction was applied only to the exploratory comparisons in Table 1 to control for multiple testing; primary hypothesis tests (ANOVA, regression, mediation) used unadjusted α = 0.05.

### 3.2. Sleep Quality Across Shift Types

Nurses in rotating three-shift schedules had the highest PSQI scores (mean = 10.2 ± 2.6), indicating poorer sleep quality than other groups.

**Figure 2:**
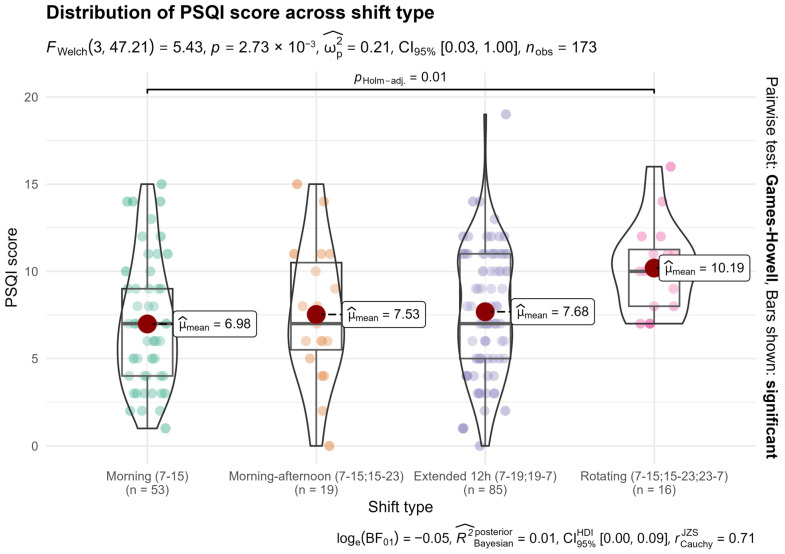
PSQI by shift type. Violin–boxplots of PSQI scores across shift schedules (higher = worse sleep). The omnibus effect size was small (ω^2^ = 0.04, 95% BCa CI [0.00, 0.09]).

A one-way Welch ANOVA showed that sleep quality differed significantly by shift type (F(3, 47.21) = 5.43, *p* = 0.003; FDR-adjusted q = 0.005; Table 1). The corresponding omnibus effect size was small (ω^2^ = 0.04, 95% BCa CI [0.00, 0.09]) (Figure 2).

Games-Howell tests showed that nurses on rotating shifts had significantly poorer sleep than all other shift types: morning (*p* = 0.002, g = 0.89), morning–afternoon (*p* < 0.05, g = 0.77), and extended 12-h schedules (*p* = 0.012, g = 0.81). No differences were observed among the three non-rotating shift types. Other Games–Howell tests were non-significant (Table S1). Visual summaries of all pairwise PSQI differences are provided in Supplementary Figure S3.

### 3.3. Physical Quality of Life

**Figure 3:**
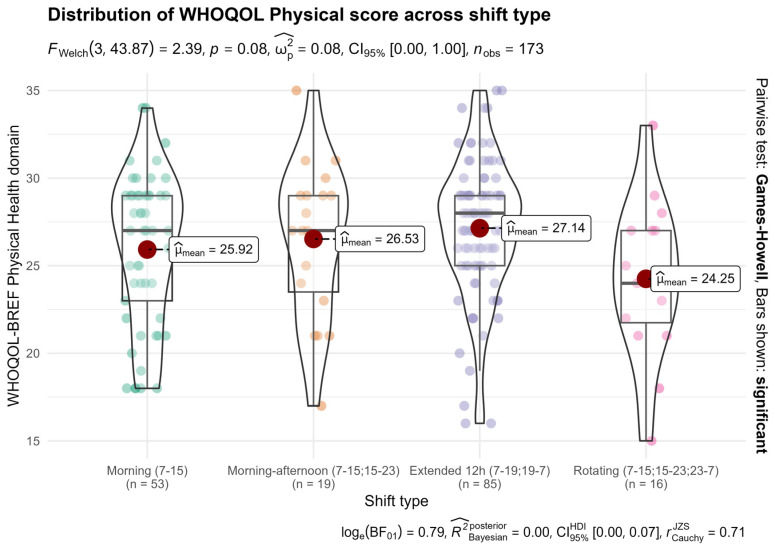
WHOQOL-BREF Physical Health by shift type. Distributions of physical quality-of-life scores by schedule, showing slightly lower values for rotating-shift nurses; differences did not reach statistical significance (*p* = 0.08).

Although mean physical well-being scores were lower among rotating-shift nurses, the between-group difference did not reach statistical significance (*p* = 0.08) (Figure 3).

A Welch ANOVA tested whether WHOQOL-BREF Physical Health scores differed by shift type. The omnibus test approached but did not reach statistical significance (F(3, 43.87) = 2.39, *p* = 0.082; ω^2^ = 0.03, 95% BCa bootstrap CI [0.00, 0.08]). Descriptive statistics (Table 1) showed that rotating-shift nurses had the lowest mean physical health scores (M = 24.3, SD = 4.4), while extended 12-h nurses had the highest (M = 27.1, SD = 4.0).

Games-Howell post hoc tests revealed no statistically significant pairwise differences at α = 0.05 (Table S2, Figure S1). The largest effect was between extended 12-h and rotating shifts (mean difference = 2.89, 95% CI [−6.19, 0.41], *p* = 0.10; Hedges’ g = 0.69, 95% CI [0.20, 1.32]), representing a medium-to-large effect size that did not reach statistical significance; the wide confidence intervals reflect limited precision, particularly for the small rotating-shift group (n = 16).

Comparisons involving morning-shift nurses showed small-to-moderate effects: morning vs. morning–afternoon (g = −0.14, *p* = 0.96), morning vs. extended 12-h (g = −0.29, *p* = 0.35), and morning vs. rotating (g = 0.38, *p* = 0.54). The morning–afternoon vs. rotating comparison showed a moderate effect (g = 0.50, 95% CI [−0.12, 1.33], *p* = 0.44).

The borderline omnibus *p*-value (0.082) and the largest pairwise effect (extended 12-h vs. rotating; g = 0.69) suggest a substantive pattern—rotating-shift nurses reporting lower physical well-being—that may be underpowered in this sample. This pattern, combined with the significant sleep quality differences (*p* = 0.003), motivated the subsequent regression and mediation analyses to test whether sleep quality was statistically consistent with the observed physical well-being differences. Forest plots of WHOQOL-BREF Physical pairwise comparisons are shown in Supplementary Figure S4.

### 3.4. Regression Analyses

In a multiple linear regression model (Table 2), rotating-shift work was associated with poorer sleep quality (higher PSQI scores) after adjusting for age, education, work experience, and on-call status (β = +3.1, *p* = 0.019).

On-call assignments vary in scheduling; without activation-time data, we avoid conclusions about their circadian impact. This counterintuitive association (on-call duties linked to lower PSQI scores) likely reflects selection mechanisms or residual confounding rather than a genuine protective effect of on-call work on sleep, and should not be interpreted causally. When on-call participants were excluded, the direction and magnitude of all other associations, including the higher PSQI scores among rotating-shift nurses, remained essentially unchanged (β = +2.05, *p* = 0.10). Education showed a modest association with lower PSQI scores, while age and experience were not significantly associated with PSQI in this model. Although model fit was modest (adjusted R^2^ = 0.07), the direction and magnitude of effects remained stable across all robustness checks.

In the multiple linear regression predicting WHOQOL-BREF Physical Health scores (Table 3), poorer sleep quality (higher PSQI) was strongly and independently associated with lower physical well-being (β = −0.63, 95% CI −0.78 to −0.48; *p* < 0.001). After adjusting for PSQI, shift type was no longer significant (overall *p* = 0.22). Longer work experience showed a negative gradient (β ≈ −1.3 to −5.0 across categories), whereas higher educational attainment showed a positive trend. Model fit improved substantially (adjusted R^2^ = 0.36), indicating that sleep quality contributed substantially to the variance explained by the regression model for physical well-being in this cross-sectional sample.

Including PSQI in the outcome model substantially attenuated the shift-type coefficients, a pattern statistically consistent with sleep quality accounting for part of the observed cross-sectional association; this motivated the subsequent mediation analysis (statistical decomposition).

### 3.5. Mediation Analysis

**Figure 4:**
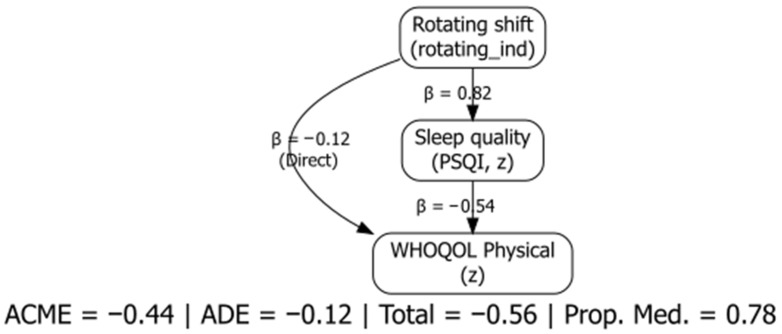
Statistical mediation model (cross-sectional decomposition) relating rotating shifts, sleep quality (PSQI), and physical well-being (WHOQOL-BREF Physical Health). Path coefficients in the diagram are standardized (β); ACME/ADE/Total reported in the text are unstandardized with BCa 95% CIs (10,000 resamples). The indirect association via sleep quality between rotating shifts and physical well-being was statistically significant (ACME = −1.85, 95% CI −3.05 to −0.88; *p* < 0.001).

Given the cross-sectional design of this survey, mediation analysis identifies statistical patterns that are consistent with, but do not demonstrate, a causal pathway. Results should therefore be interpreted as indicative of indirect associations rather than proof of temporal or causal influence. The mediation model tested whether sleep quality (PSQI) was statistically consistent with an indirect association between rotating shifts and physical quality of life. Bootstrapped mediation (10,000 resamples, BCa method) indicated a statistically significant indirect association via sleep quality (ACME = −1.85, 95% CI −3.05 to −0.88; *p* < 0.001). The average direct effect was non-significant (ADE = −0.52, 95% CI −2.64 to 1.63; *p* = 0.60), yielding a small total effect of −2.38 (95% CI −4.76 to −0.12; *p* = 0.046). Because higher PSQI indicates poorer sleep, the negative ACME reflects the pattern that lower physical scores among rotating-shift nurses were statistically consistent with an indirect association via worse sleep quality (Figure 4).

Sensitivity analysis showed that the indirect effect via sleep quality remained statistically significant for residual correlations up to |ρ| ≤ 0.5. Equivalently, an unmeasured confounder would need to explain approximately 13% of the residual variance in both the mediator and outcome models (R^2^_M ≈ R^2^_Y ≈ 0.13), or about 25% jointly, to attenuate the indirect association statistically consistent with sleep quality to zero (Figure S2; Table S5).

### 3.6. Model Diagnostics

Residual diagnostics (Q–Q and residual–fitted plots) supported acceptable approximation of linear model assumptions. Although Shapiro–Wilk indicated non-normality for WHOQOL (*p* = 0.001), linear models are reasonably robust at N ≈ 173; we therefore complemented all key estimates with bootstrap CIs.

## 4. Discussion

This study had two objectives: (1) to compare sleep quality and physical well-being (WHOQOL-BREF Physical Health domain) across four shift schedules among hospital nurses and (2) to examine whether associations between shift type and physical well-being were statistically consistent with an indirect association via sleep quality. Rotating-shift nurses reported significantly poorer sleep than all other shift groups, whereas physical well-being differences by shift type did not reach statistical significance. In the mediation framework, rotating-shift schedules showed a statistically significant indirect association with lower physical well-being via poorer sleep, while the direct association was small and imprecise.

Taken together, these findings indicate that poorer sleep quality was statistically associated with most of the observed relationship between rotating shifts and physical well-being in this sample. However, the modest total association, the small rotating-shift subgroup, and the cross-sectional, self-report design mean that these results are hypothesis-generating rather than causal; accordingly, estimates involving rotating shifts are imprecise and accompanied by wide confidence intervals. The mediation analysis provides a statistical decomposition of the observed association into an indirect component statistically consistent with differences in sleep quality and a remaining component not captured by sleep quality; it does not establish temporal ordering or exclude unmeasured confounding. The disappearance of the direct shift-type term once sleep quality was included suggests that sleep differences may statistically account for much of the association between rotating schedules and physical well-being, although the proportion of the association attributed to the indirect component was imprecisely estimated and requires confirmation in larger, longitudinal studies.

Our findings align with prior evidence showing that rotating and night-including schedules are associated with poorer sleep quality and higher burden of shift-work disorder among nurses [7,8,16]. The pattern observed here—poorer sleep on rotating schedules and lower physical quality of life—is consistent with earlier reports of higher PSQI scores, more fatigue-related problems, and lower quality-of-life scores in shift-working nurses compared with day workers [7,8,16]. Converging literature also links shift exposure to adverse metabolic profiles in healthcare workers, including higher body-mass index, fasting glucose, and blood pressure values in nurses working rotating night shifts [10,17]. In some cohorts, the frequency and duration of rotating night work correlate with more unfavorable metabolic and cardiovascular risk markers, suggesting a possible metabolic burden alongside sleep disruption [17].

Recent work has further connected poor sleep quality among rotating-shift nurses with higher rates of burnout, depressive symptoms, and compassion fatigue, highlighting the psychological as well as physical dimensions of circadian disruption [18]. In our regression models, longer work experience showed a negative gradient in physical quality of life, consistent with the idea that cumulative occupational strain may exert a stronger effect on physical well-being than current shift pattern alone. Prior studies have examined whether sleep is associated with the relationship between shift schedules and health outcomes, with mixed findings across endpoints and designs [10]. Within this context, our cross-sectional mediation analysis should be interpreted as a statistical decomposition that is consistent with—but does not prove—a pathway via degraded sleep quality.

A notable and counterintuitive finding was that on-call duty was associated with better sleep scores in the regression model. This likely reflects selection mechanisms (e.g., on-call roles allocated to more experienced or higher-functioning staff) or residual confounding, rather than any protective effect of on-call work on sleep. We therefore treated on-call status as a covariate and do not interpret this association causally.

This study has several strengths. We used widely applied, validated instruments (PSQI and WHOQOL-BREF Physical Health), conducted all analyses using an openly shared script, and applied bootstrapped confidence intervals with extensive diagnostics to quantify uncertainty. All data and code are publicly available, supporting reproducibility. The focus on a real-world tertiary hospital sample and detailed shift classification provides clinically interpretable estimates for a common staffing pattern in hospital nursing.

### Limitations

This was a single-center study from one Croatian tertiary hospital; recruitment relied on voluntary participation with a response rate of approximately 39%, which may introduce selection bias, and results may not generalize to other institutions, countries, or non-hospital settings. Nurses experiencing greater fatigue, poorer sleep, or more demanding schedules may have been less likely to participate, although the direction and magnitude of any non-response bias cannot be determined from the available data. The analysis focused exclusively on the WHOQOL-BREF Physical Health domain to provide a clear, physiologically grounded outcome; other WHOQOL domains were not analyzed and may capture additional aspects of well-being. The study relied on cross-sectional, self-reported data, precluding causal inference and making the mediation analysis a purely statistical decomposition of associations rather than proof of temporal pathways. Unmeasured confounders such as lifestyle factors, comorbidities, or family obligations may have contributed to residual variance.

A primary limitation is the small rotating-shift subgroup (n = 16), which reduces the precision of estimates for this group. This distribution reflects typical staffing structures in Croatian hospitals, where rotating night duties are concentrated among a limited subset of nurses rather than evenly distributed. We used nonparametric bootstrapping and sensitivity analyses to better quantify uncertainty under unequal group sizes, but these approaches cannot fully overcome the instability inherent in a very small rotating-shift subgroup; confidence intervals remain wide and effect magnitudes should be interpreted cautiously. Finally, longitudinal nurse cohorts have shown that changes in night-work exposure predict subsequent sleep problems and shift-work disorder [19]; future research should therefore use prospective designs with refined exposure histories to test whether the patterns observed here hold in larger, multi-center samples.

## 5. Conclusions

This cross-sectional study found patterns statistically consistent with an association between rotating night shifts and poorer sleep quality, with lower physical well-being on average among rotating-shift nurses. Rotating-shift nurses reported significantly poorer sleep (PSQI = 10.2 ± 2.6; *p* = 0.003), whereas physical well-being scores were lower on average but did not differ significantly across shift types (*p* = 0.082). Within a bootstrapped mediation analysis (interpreted as a statistical decomposition), the indirect association statistically consistent with sleep quality was significant (ACME = −1.85, 95% CI −3.05 to −0.88; *p* < 0.001), while the direct component was non-significant and imprecise; however, the rotating-shift subgroup was small (n = 16) and the proportion attributable to the indirect component was therefore imprecisely estimated.

If future longitudinal and experimental research confirms that changes in sleep quality form part of a causal pathway between rotating night work and physical health, this would support prioritizing sleep-restorative and chronoprotective scheduling strategies—such as limiting consecutive night shifts, ensuring sufficient recovery periods, supporting structured sleep hygiene education, improving rest facilities, and accounting for chronotype preferences—within occupational health policy. At present, however, the data remains cross-sectional and self-reported; the mediation results should be interpreted as statistical consistency with a sleep-related pathway rather than proof of causal mediation. Prospective, multi-center studies using objective circadian and sleep measures (e.g., actigraphy, melatonin profiling) and randomized scheduling or sleep interventions are needed to test whether the patterns observed here reflect true causal mechanisms.

## Figures and Tables

**Table 1:** Participant Characteristics by Shift Type.

Variable	Overall N = 173	Morning (7–15) N = 53	Morning–Afternoon (7–15; 15–23) N = 19	Extended 12-h (7–19; 19–7) N = 85	Rotating (7–15; 15–23; 23–7) N = 16	*p*-Value ^1^	q-Value ^2^
Age, Mean (SD)	36.4 (11.8)	42.1 (11.7)	44.3 (10.4)	31.3 (9.8)	34.9 (10.4)	<0.001	<0.001
Sex, n (%)						0.080	0.089
Male	28 (16%)	7 (13%)	1 (5.3%)	14 (16%)	6 (38%)		
Female	145 (84%)	46 (87%)	18 (95%)	71 (84%)	10 (63%)		
Education, n (%)						0.089	0.089
Secondary	67 (39%)	15 (28%)	4 (21%)	40 (47%)	8 (50%)		
Bachelor	91 (53%)	31 (58%)	14 (74%)	38 (45%)	8 (50%)		
Master	15 (8.7%)	7 (13%)	1 (5.3%)	7 (8.2%)	0 (0%)		
Work Experience, n (%)						<0.001	<0.001
≤5	45 (26%)	4 (7.5%)	1 (5.3%)	37 (44%)	3 (19%)		
6–15	55 (32%)	16 (30%)	4 (21%)	28 (33%)	7 (44%)		
16–25	34 (20%)	12 (23%)	7 (37%)	12 (14%)	3 (19%)		
26–35	24 (14%)	13 (25%)	3 (16%)	6 (7.1%)	2 (13%)		
>35	15 (8.7%)	8 (15%)	4 (21%)	2 (2.4%)	1 (6.3%)		
PSQI score, Mean (SD)	7.7 (3.6)	7.0 (3.6)	7.5 (3.9)	7.7 (3.5)	10.2 (2.6)	0.003	0.005
WHOQOL-BREF Physical Health domain, Mean (SD)	26.4 (4.2)	25.9 (4.3)	26.5 (4.4)	27.1 (4.0)	24.3 (4.4)	0.082	0.089

^1^ Welch’s ANOVA; Fisher’s exact test; Pearson’s Chi-squared test. ^2^ False discovery rate correction for multiple testing.

**Table 2:** Linear regression predicting PSQI score.

Characteristic	Beta (95% CI)	*p*-Value
(Intercept)	7.7 (4.1, 11)	<0.001
Shift type		0.019
Morning (7–15)	—	
Morning–afternoon (7–15; 15–23)	−0.08 (−2.0, 1.9)	
Extended 12-h (7–19; 19–7)	0.26 (−1.2, 1.7)	
Rotating (7–15; 15–23; 23–7)	3.1 (1.1, 5.1)	
Age (years)	0.00 (−0.12, 0.13)	0.96
Education level		0.18
Secondary	—	
Bachelor	−0.83 (−2.0, 0.34)	
Master	−1.7 (−3.8, 0.34)	
Work experience (years)		0.79
≤5	—	
6–15	−0.17 (−1.8, 1.5)	
16–25	0.68 (−1.9, 3.3)	
26–35	1.2 (−2.6, 5.0)	
>35	0.28 (−4.4, 5.0)	
on call		0.037
no	—	
yes	−2.0 (−4.0, −0.13)	

Abbreviation: CI = Confidence Interval. R^2^ = 0.131; Adjusted R^2^ = 0.072; No. Obs. = 173.

**Table 3:** Linear regression predicting WHOQOL-BREF Physical Health score.

Characteristic	Beta (95% CI)	*p*-Value
Shift type		0.22
Morning (7–15)	—	
Morning–afternoon (7–15; 15–23)	1.3 (−0.57 to 3.2)	
Extended 12-h (7–19; 19–7)	1.4 (−0.03 to 2.8)	
Rotating (7–15; 15–23; 23–7)	0.32 (−1.7 to 2.3)	
PSQI	−0.63 (−0.78 to −0.48)	<0.001
Age (years)	0.07 (−0.05 to 0.19)	0.25
Education level		0.47
Secondary	—	
Bachelor	0.52 (−0.63 to 1.7)	
Master	1.1 (−0.89 to 3.2)	
Work experience (years)		0.26
≤5	—	
6–15	−1.3 (−2.9 to 0.30)	
16–25	−2.7 (−5.2 to −0.20)	
26–35	−3.4 (−7.1 to 0.33)	
>35	−5.0 (−9.6 to −0.43)	
On call		0.38
No	—	
Yes	0.84 (−1.1 to 2.7)	

Abbreviation: CI = Confidence Interval. R^2^ = 0.406; Adjusted R^2^ = 0.362; No. Obs. = 173.

## Data Availability

The original data presented in the study are openly available in OSF at https://doi.org/10.17605/OSF.IO/8HCXU.

## Supplementary Materials

The following supporting information can be downloaded at https://www.mdpi.com/article/10.3390/nursrep16010019/s1, Table S1: Games–Howell pairwise comparisons for PSQI by shift type; Table S2: Games–Howell pairwise comparisons for WHOQOL-Bref Physical by shift type; Table S3: Reliability of scales (Cronbach’s α with 95% CI); Table S4: Standardized coefficients for mediator and outcome models; Table S5: Sensitivity analysis excluding on-call staff; Figure S1: Diagnostic plots for regression models; Figure S2: Forest plot of pairwise PSQI differences (Games–Howell comparisons); Figure S3: Forest plot of pairwise WHOQOL-BREF Physical Health differences (Games–Howell comparisons). Figure S4: Forest Plot of Pairwise WHOQOL-BREF Physical Health Differences (Games–Howell comparisons).

## Public Involvement Statement

There was no public involvement in any aspect of this research.

## Guidelines and Standards Statement

This manuscript was drafted in accordance with the Strengthening the Reporting of Observational Studies in Epidemiology (STROBE) Statement (cross-sectional checklist) for observational cross-sectional research [20].

## Abbreviations

The following abbreviations are used in this manuscript:

PSQI	Pittsburgh Sleep Quality Index
STROBE	Strengthening the Reporting of Observational Studies in Epidemiology
VIF	Variance Inflation Factor
WHOQOL-BREF	World Health Organization Quality of Life (WHOQOL)-BREF

## References

[1] Rosa D., Terzoni S., Dellafiore F., Destrebecq A. (2019). Systematic review of shift work and nurses’ health. Occup. Med..

[2] Boniol M., Kunjumen T., Nair T.S., Siyam A., Campbell J., Diallo K. (2022). The global health workforce stock and distribution in 2020 and 2030: A threat to equity and ‘universal’ health coverage?. BMJ Glob. Health.

[3] Ruiz-Fernandez M.D., Perez-Garcia E., Ortega-Galan A.M. (2020). Quality of Life in Nursing Professionals: Burnout, Fatigue, and Compassion Satisfaction. Int. J. Environ. Res. Public Health.

[4] Razavi P., Devore E.E., Bajaj A., Lockley S.W., Figueiro M.G., Ricchiuti V., Gauderman W.J., Hankinson S.E., Willett W.C., Schernhammer E.S. (2019). Shift Work, Chronotype, and Melatonin Rhythm in Nurses. Cancer Epidemiol. Biomark. Prev..

[5] Membrive-Jimenez M.J., Gomez-Urquiza J.L., Suleiman-Martos N., Velando-Soriano A., Ariza T., De la Fuente-Solana E.I., Canadas-De la Fuente G.A. (2022). Relation between Burnout and Sleep Problems in Nurses: A Systematic Review with Meta-Analysis. Healthcare.

[6] Flo E., Pallesen S., Mageroy N., Moen B.E., Gronli J., Hilde Nordhus I., Bjorvatn B. (2012). Shift work disorder in nurses--assessment, prevalence and related health problems. PLoS ONE.

[7] Lin P.C., Chen C.H., Pan S.M., Pan C.H., Chen C.J., Chen Y.M., Hung H.C., Wu M.T. (2012). Atypical work schedules are associated with poor sleep quality and mental health in Taiwan female nurses. Int. Arch. Occup. Environ. Health.

[8] Palhares Vde C., Corrente J.E., Matsubara B.B. (2014). Association between sleep quality and quality of life in nursing professionals working rotating shifts. Rev. Saude Publica.

[9] Di Muzio M., Diella G., Di Simone E., Novelli L., Alfonsi V., Scarpelli S., Annarumma L., Salfi F., Pazzaglia M., Giannini A.M. (2020). Nurses and Night Shifts: Poor Sleep Quality Exacerbates Psychomotor Performance. Front. Neurosci..

[10] Lajoie P., Aronson K.J., Day A., Tranmer J. (2015). A cross-sectional study of shift work, sleep quality and cardiometabolic risk in female hospital employees. BMJ Open.

[11] Dall’Ora C., Griffiths P., Ball J., Simon M., Aiken L.H. (2015). Association of 12 h shifts and nurses’ job satisfaction, burnout and intention to leave: Findings from a cross-sectional study of 12 European countries. BMJ Open.

[12] Dong H., Zhang Q., Zhu C., Lv Q. (2020). Sleep quality of nurses in the emergency department of public hospitals in China and its influencing factors: A cross-sectional study. Health Qual. Life Outcomes.

[13] Chang W.P., Peng Y.X. (2021). Influence of rotating shifts and fixed night shifts on sleep quality of nurses of different ages: A systematic literature review and meta-analysis. Chronobiol. Int..

[14] Buysse D.J., Reynolds C.F., Monk T.H., Berman S.R., Kupfer D.J. (1989). The Pittsburgh Sleep Quality Index: A new instrument for psychiatric practice and research. Psychiatry Res..

[15] Group W. (1998). Development of the World Health Organization WHOQOL-BREF quality of life assessment. Psychol. Med..

[16] Gander P., O’Keeffe K., Santos-Fernandez E., Huntington A., Walker L., Willis J. (2019). Fatigue and nurses’ work patterns: An online questionnaire survey. Int. J. Nurs. Stud..

[17] Qiao H., Beibei Z., Chong T., Tieying Z., Yuzhi G., Jing M., Davidson P.M. (2020). Both frequency and duration of rotating night shifts are associated with metabolic parameters: A cross-sectional study. Sleep Med..

[18] Al-Hammouri M.M., Rababah J., Al-Jdeetawey N.A. (2025). How Shift Schedules Shape Nurses’ Sleep and Compassion: A Comparative Study. Int. Nurs. Rev..

[19] Waage S., Pallesen S., Moen B.E., Mageroy N., Flo E., Di Milia L., Bjorvatn B. (2014). Predictors of shift work disorder among nurses: A longitudinal study. Sleep Med..

[20] von Elm E., Altman D.G., Egger M., Pocock S.J., Gotzsche P.C., Vandenbroucke J.P., Initiative S. (2007). The Strengthening the Reporting of Observational Studies in Epidemiology (STROBE) statement: Guidelines for reporting observational studies. PLoS Med..

