# Supplementary material for: Rotating Night Shifts and Physical Well-Being in Nurses: Cross-Sectional Associations Consistent with a Sleep Quality Pathway
Source: Nurs Rep. 2026 Jan 8;16(1):19. doi: 10.3390/nursrep16010019 (PMC12845087; doi:10.3390/nursrep16010019)
Supplement: Supplementary file 1 [file nursrep-16-00019-s001.zip › nursrep-4004037-supplementary.pdf]

Supplementary Materials

Rotating Night Shifts and Physical Well-Being in Nurses: Cross-Sectional Associations Consistent with a Sleep-Quality Pathway

Table S1. Games–Howell Pairwise Comparisons for PSQI by Shift Type

Group 1	Group 2	Mean difference	Adjusted p-value	95% CI	Hedges' g	95% CI
Morning (7-15)	Morning-afternoon (7-15;15-23)	0.55	0.95	[-2.22, 3.31]	−0.14	[-0.72, 0.44]
Morning (7-15)	Extended 12h (7-19;19-7)	0.70	0.68	[-0.93, 2.33]	−0.20	[-0.56, 0.14]
Morning (7-15)	Rotating (7-15;15-23;23-7)	3.21	<0.01	[1.03, 5.39]	−1.02	[-1.59, -0.54]
Morning-afternoon (7-15;15-23)	Extended 12h (7-19;19-7)	0.16	1.00	[-2.51, 2.82]	−0.04	[-0.59, 0.47]
Morning-afternoon (7-15;15-23)	Rotating (7-15;15-23;23-7)	2.66	0.09	[-0.31, 5.63]	−0.79	[-1.55, -0.14]
Extended 12h (7-19;19-7)	Rotating (7-15;15-23;23-7)	2.51	0.01	[0.46, 4.55]	−0.80	[-1.31, -0.36]

Note: p-values are adjusted using the Games–Howell procedure. Confidence intervals for Hedges' g are unadjusted and are provided for descriptive interpretation of effect magnitude only. Comparisons involving the rotating shift group should be interpreted cautiously due to small subgroup size.

Table S2. Games–Howell Pairwise Comparisons for WHOQOL-BREF Physical Health Domain by Shift Type (N = 173)

Group 1	Group 2	Mean difference	Adjusted p-value	95% CI	Hedges’ *g*	95% CI
Morning (7-15)	Morning-afternoon (7-15;15-23)	0.60	0.96	[-2.59, 3.79]	−0.14	[-0.68, 0.40]
Morning (7-15)	Extended 12h (7-19;19-7)	1.22	0.35	[-0.69, 3.12]	−0.29	[-0.64, 0.06]
Morning (7-15)	Rotating (7-15;15-23;23-7)	−1.67	0.54	[-5.11, 1.76]	0.38	[-0.18, 1.05]
Morning-afternoon (7-15;15-23)	Extended 12h (7-19;19-7)	0.61	0.94	[-2.43, 3.66]	−0.15	[-0.65, 0.42]
Morning-afternoon (7-15;15-23)	Rotating (7-15;15-23;23-7)	−2.28	0.44	[-6.33, 1.78]	0.50	[-0.12, 1.33]
Extended 12h (7-19;19-7)	Rotating (7-15;15-23;23-7)	−2.89	0.10	[-6.19, 0.41]	0.69	[0.20, 1.31]

Note: p-values are adjusted using the Games–Howell procedure. Confidence intervals for Hedges’ g are unadjusted and are provided for descriptive interpretation of effect magnitude only. Comparisons involving the rotating shift group should be interpreted cautiously due to small subgroup size.

Table S3. Reliability of scales (Cronbach's α with 95 % CI)

scale	Number of items	Complete cases (n)	Cronbach's α (raw)	Cronbach's α (standardized)	Average inter-item correlation	α 95% CI
PSQI	7	173	0.684	0.697	0.247	0.603 - 0.747
WHOQOL-BREF Physical	7	173	0.806	0.811	0.380	0.753 - 0.845

Note: Reliability estimates based on complete-case data (n = 173). Confidence intervals derived by 10,000-sample bootstrapping. The PSQI showed borderline internal consistency, consistent with prior studies reporting moderate internal consistency for this instrument.

Table S4. Standardized coefficients (β) with 95% confidence intervals from multivariable regression models for PSQI (sleep quality) and WHOQOL-Physical. Abbreviations: CI = confidence interval.

	PSQI model (sleep quality)		WHOQOL-Physical model	
	Standardized β (contrast vs reference)			
Predictor	β (95% CI)	p	β (95% CI)	p
Age (years)	0.00 (-0.41, 0.41)	0.99	0.20 (-0.14, 0.54)	0.25
Education: Bachelor vs Secondary	-0.25 (-0.56, 0.07)	0.13	0.10 (-0.17, 0.37)	0.45
Education: Master vs Secondary	-0.50 (-1.06, 0.07)	0.09	0.20 (-0.28, 0.68)	0.40
Experience: 16–25 vs ≤5	0.17 (-0.54, 0.87)	0.64	-0.72 (-1.31, -0.13)	0.02
Experience: 26–35 vs ≤5	0.31 (-0.73, 1.35)	0.56	-0.90 (-1.78, -0.03)	0.04
Experience: 6–15 vs ≤5	-0.06 (-0.51, 0.40)	0.81	-0.34 (-0.72, 0.04)	0.08
Experience: >35 vs ≤5	0.04 (-1.25, 1.33)	0.95	-1.32 (-2.41, -0.24)	0.02
On-call (1 = yes)	-0.59 (-1.08, -0.10)	0.02	0.03 (-0.39, 0.44)	0.90
Rotating (1 = yes)	0.24 (0.09, 0.39)	<0.01	-0.04 (-0.16, 0.09)	0.58
Sleep quality (PSQI)	Not applicable	Not applicable	-0.54 (-0.67, -0.41)	<0.01

Note: Estimates from ordinary least-squares regression with variables standardized via refitting; 95 % confidence intervals computed parametrically. Standardized coefficients for categorical predictors represent differences relative to the reference category expressed in standard deviation units of the outcome. Coefficients represent adjusted cross-sectional associations and should not be interpreted as causal or temporal effects.

Table S5. Sensitivity analysis excluding on-call staff (n = 19 excluded): standardized regression coefficients (β) with 95% confidence intervals

Model	Predictor	β	95 % CI	p
PSQI model	Rotating vs Morning	2.05	(–0.39, 4.48)	0.10
WHOQOL model	PSQI total	–0.65	(–0.80, –0.50)	<0.01

Note: Models were specified identically to those in Table S4, excluding participants with on-call duties (n = 19). Effect estimates were comparable in magnitude and direction to the main analysis. Patterns of effects remained stable across models.

Figure S1. Diagnostic plots for regression models: Panels A–B show residuals vs fitted values and Q–Q plots for the PSQI model (sleep quality; rotating + covariates). Panels C–D show corresponding diagnostics for the WHOQOL-Physical model (PSQI + rotating + covariates). Visual inspection indicated acceptable approximation to linear regression assumptions, with no evidence of influential observations.

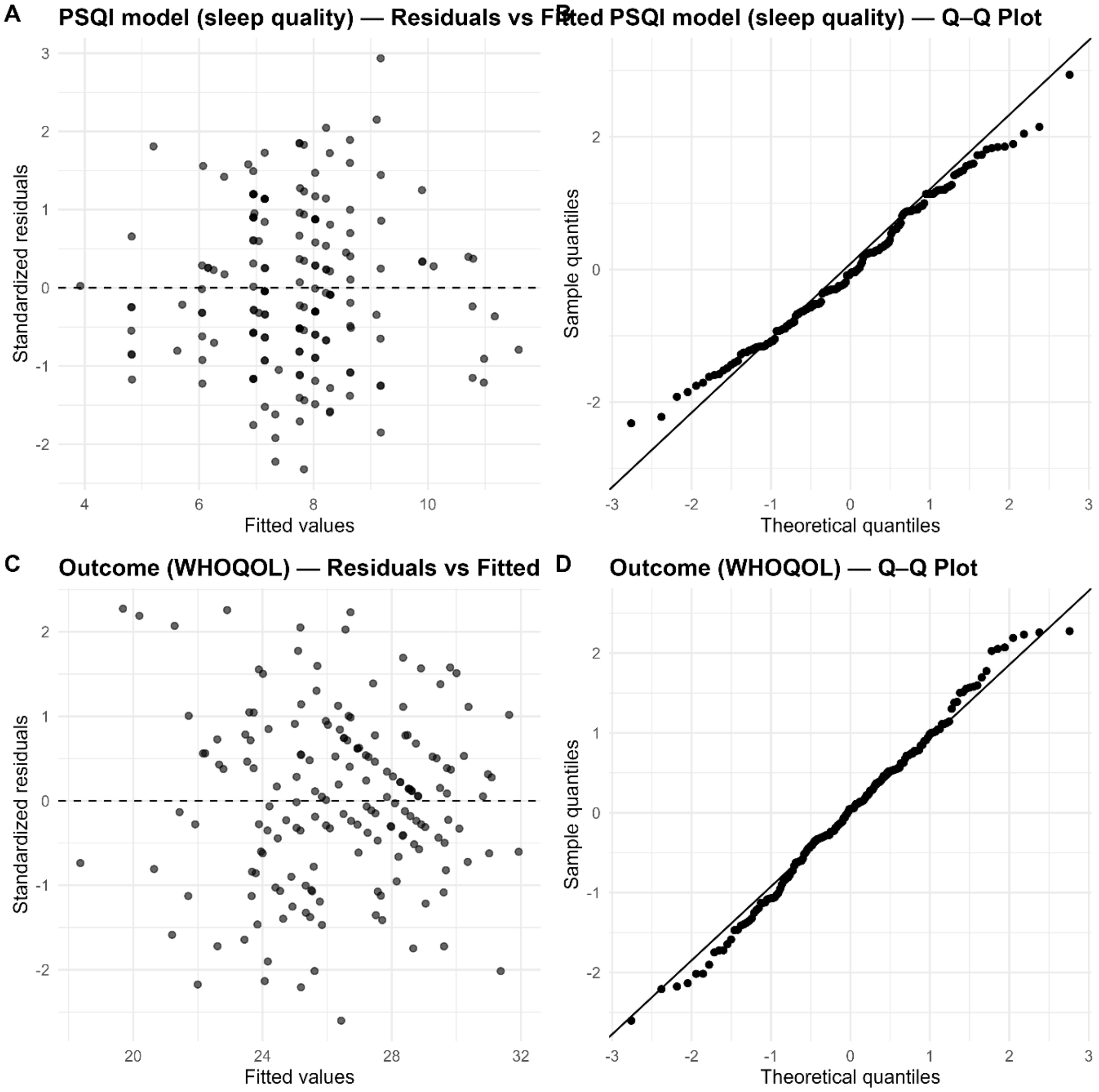

Figure S2. Exploratory sensitivity analysis under hypothetical causal mediation assumptions. The solid line shows the Average Causal Mediation Effect (ACME) as a function of the residual correlation (ϱ) between the mediator and outcome models, as implemented in the medsens function of the R mediation package. The shaded area denotes the corresponding 95% confidence interval. This analysis assumes a correctly specified causal mediation structure and absence of exposure–mediator interaction, assumptions that cannot be verified in the present cross-sectional study. Accordingly, the figure is presented for illustrative purposes only and does not constitute evidence of causal mediation. The plot indicates the degree of unmeasured confounding that would be required to null the ACME under these assumptions.

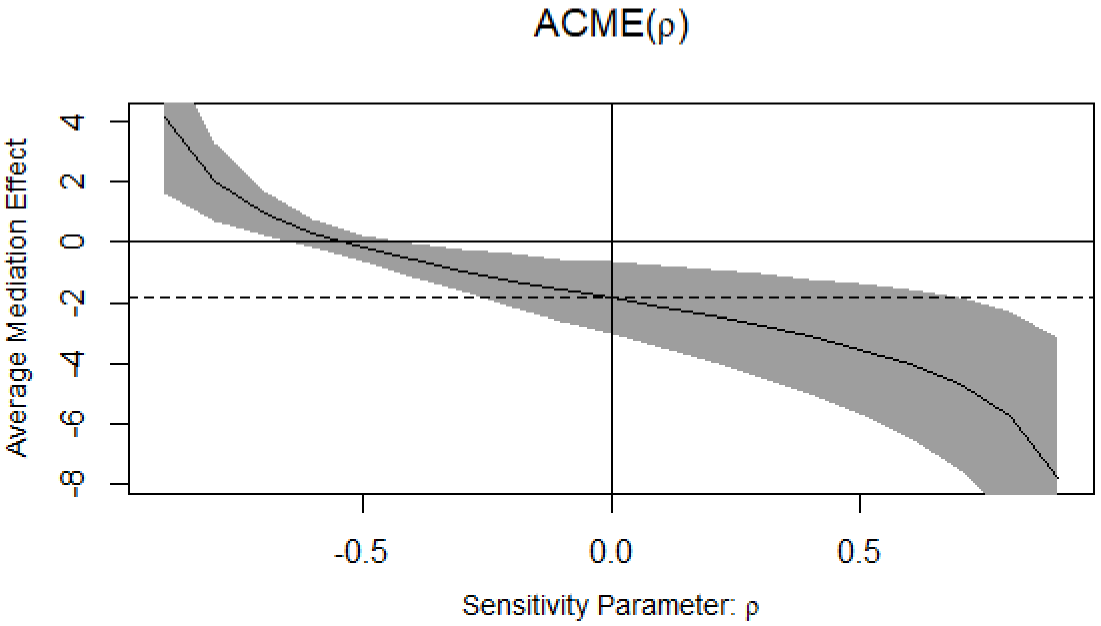

Sensitivity metrics derived from an exploratory causal mediation sensitivity analysis (corresponding to Figure S2).

Metric	Estimate
ϱ at which ACME = 0	–0.5
ACME significant range	
R^2_M × R^2_Y required to null ACME	0.25
Balanced confounding R^2_M ≈ R^2_Y	0.13

Note: All quantities are defined within a hypothetical causal mediation framework and are derived from the medsens sensitivity analysis. These metrics describe how strong unmeasured confounding between the mediator and outcome would need to be to null the ACME if the assumed causal mediation model were correctly specified. Given the cross-sectional design and lack of established temporal ordering, these values should not be interpreted as evidence for or against causal mediation.

Figure S3: Forest plot of pairwise differences in PSQI scores by shift type.
Points represent adjusted mean differences, and horizontal lines indicate 95% confidence intervals from Games–Howell post hoc comparisons. Values greater than zero indicate higher PSQI scores in the first-listed group. Statistical significance is based on Games–Howell–adjusted p-values. The figure is provided for visual comparison and complements the numerical results presented in Table S1. Note: Points are colored to indicate Games–Howell–adjusted statistical significance (red: $p < 0.05$; orange: $p \geq 0.05$ and < 0.10; black: $p \geq 0.10$).

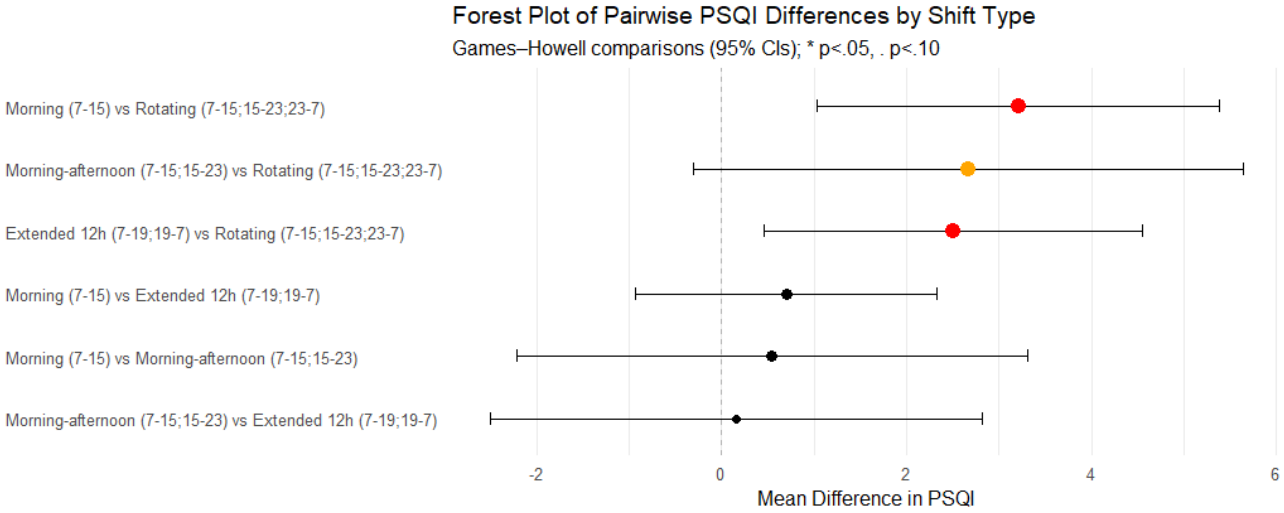

Figure S4: Forest Plot of Pairwise differences in WHOQOL-BREF Physical Health scores by shift type. Points represent adjusted mean differences, and horizontal lines indicate 95% confidence intervals from Games–Howell post hoc comparisons. Values greater than zero indicate higher WHOQOL-BREF Physical Health scores in the first-listed group. Statistical significance is based on Games–Howell–adjusted p-values. The figure is provided for visual comparison and complements the numerical results presented in Table S2. Note: Points are colored to indicate Games–Howell–adjusted statistical significance (red: $p < 0.05$; orange: $p \geq 0.05$ and < 0.10; black: $p \geq 0.10$).

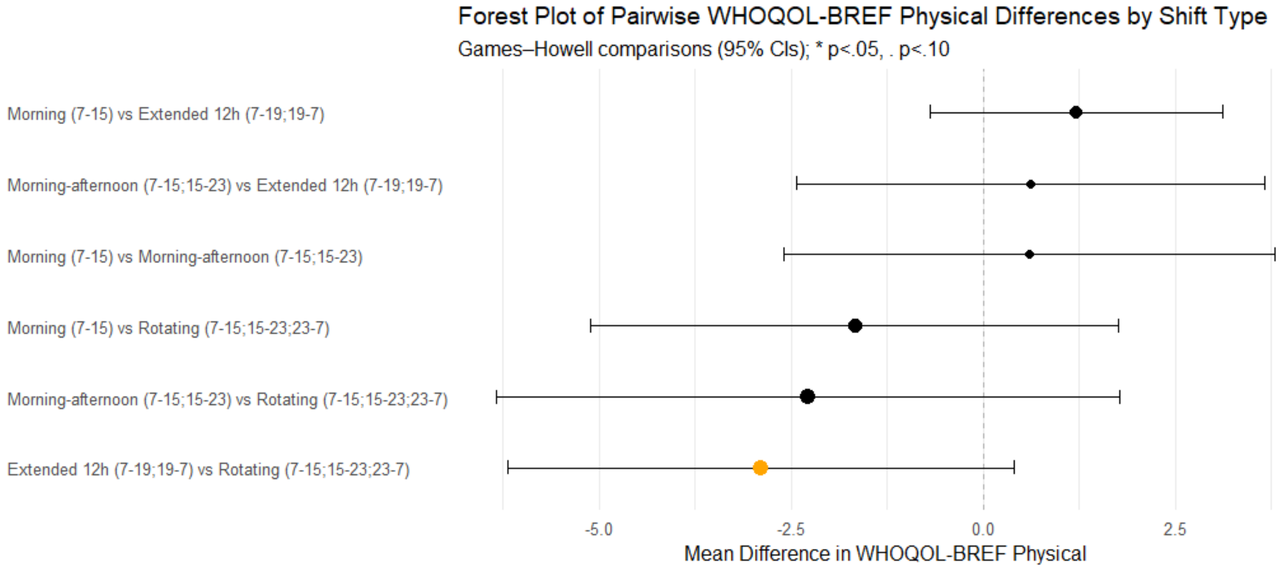

Data and R scripts are publicly available at OSF (DOI: 10.17605/OSF.IO/8HCXU).
